# Cumulative temporal vegetation indices from unoccupied aerial systems allow maize (*Zea mays* L.) hybrid yield to be estimated across environments with fewer flights

**DOI:** 10.1371/journal.pone.0277804

**Published:** 2023-01-26

**Authors:** Sumantra Chatterjee, Alper Adak, Scott Wilde, Shakirah Nakasagga, Seth C. Murray

**Affiliations:** 1 Department of Soil and Crop Sciences, Texas A&M University, College Station, TX, United States of America; 2 Department of Horticulture, University of Wisconsin, Madison, Wisconsin, United States of America; Universidade do Vale do Rio dos Sinos, BRAZIL

## Abstract

Unoccupied aerial systems (UAS) based high throughput phenotyping studies require further investigation to combine different environments and planting times into one model. Here 100 elite breeding hybrids of maize (*Zea mays* L.) were evaluated in two environment trials–one with optimal planting and irrigation (IHOT), and one dryland with delayed planting (DHOT). RGB (Red-Green-Blue) based canopy height measurement (CHM) and vegetation indices (VIs) were estimated from a UAS platform. Time series and cumulative VIs, by both summation (ΣVI-SUMs) and area under the curve (ΣVI-AUCs), were fit via machine learning regression modeling (random forest, linear, ridge, lasso, elastic net regressions) to estimate grain yield. VIs were more valuable predictors of yield to combine different environments than CHM. Time series VIs and CHM produced high accuracies (~68–72%), but inconsistent models. A little sacrifice in accuracy (~60–65%) produced consistent models using ΣVI-SUMs and CHM during pre-reproductive vegetative growth. Absence of VIs produced poorer accuracies (by about ~5–10%). Normalized difference type VIs produced maximum accuracies, and flowering times were the best times for UAS data acquisition. This study suggests that the best yielding varieties can be accurately predicted in new environments at or before flowering when combining multiple temporal flights and predictors.

## Introduction

Unoccupied aerial systems (UAS) have become an important tool in high throughput phenotyping studies [1–6]. UAS compared to satellite images have smaller field of view (FOV) and smaller altitude of data acquisition, and thus higher resolution, but lower aperture. For UAS to cover an entire agricultural field, multiple images are orthomosaicked to create a single image [6]. With improved accuracy of phenotyping [7], numerous crop traits and with reduced evaluation times, UAS based methods are being used for crop yield estimation [2, 3, 8–12]. The most prominent crop phenological signatures (traits) used in high throughput phenotyping, can be grouped into morphological and physiological traits ([1]. One commonly used morphological trait used as predictor to estimate crop yield in some crops and environments, is crop or canopy height measurements (CHM) [10, 13–16]. Canopy height can be estimated via remote sensing either using LiDAR [14, 17, 18], or using image-based point cloud estimation [10, 16, 19, 20]. Alternatively, vegetation indices (VIs) (s for plural), which indicate crop health and/or structural growth, are used as a proxy for crop physiological traits and to estimate yield [21–26]. The green leaves from healthy plants reflect near-infrared (NIR) and green (G) light and absorb red (R) and blue (B) light. As plants health deteriorate and greenness decreases, plants begin to reflect more blue and red lights [27]. Vegetation indices are intended to amplify these differences between high and low reflectance of crop canopies via different mathematical formulas so that they become indicator of plant’s health conditions and/or growth stages [28]. Unlike raw spectral values, VIs have an advantage of not requiring spectral correction.

When satellite images (data) are used, Normalized Difference Vegetation Index (NDVI) has served as a good predictor of yield for numerous crops at landscape scale [21, 29, 30]. However, when UAS derived VIs have been used, especially in small plots and comparing varieties or treatments, other VIs have appeared as good predictors. For example, commonly selected UAS derived VIs that have been good predictors, aside from NDVI [22, 31], include Green-Normalized Difference Vegetation Index (GNDVI) [2, 11, 22, 31, 32], Visible Atmospherically Resistant Index (VARI) [33–35], and Normalized Difference Red Edge (NDRE) [11, 22, 36]. Varied modeling procedures for incorporating and evaluating different VIs in yield prediction have been reported, including hierarchical linear regression [29], multiple linear regression [22, 33], multivariate regression modeling with other phenological matrices [21], random forest [11, 32, 37], support vector machine [32], and boosted regression tree [30].

From such varied approaches used, there has been no clear consensus regarding the best VI(s) as predictors or any one best modeling technique. Comparisons are difficult because varieties, field environments, equipment, days of flight among other factors can vary. Comparison of approaches is made more challenging in that seasonal variant VIs and CHM are time series [8], while yield (estimated after harvest) is a cumulative effect of crop’s physiological stages and biomass accumulation over the entire season [38, 39]. Thus, when time series VIs are used as predictors of yield, the predictors and response belong to two different time scales. This remains a gap, that has been addressed here.

Cumulative vegetation indices (ΣVIs) are a potentially more robust alternative for resolving the ambiguities caused by differences in time frames between time series predictors and year-end yield. ΣVIs, which may serve as a proxy to total dry matter accumulated over time, have appeared to be good predictors for wheat, over semi-arid and arid agro-ecosystems of Syria [40], or over crop fields covering large regions in Montana [41], two adjacent vineyards in California [42], maize over entire Switzerland [43], and a mixed vegetation over “40 census agricultural region” in Canada [44]. All such reported previous studies, to our knowledge, used satellite derived vegetation indices as predictors of yield. We are unaware of ΣVIs being previously tested with UAS based RGB images. Regardless of it only ΣVIs based models may be less predictive within an environment, they likely have advantages over time series models when combining different environments and different flight dates.

Aside from determining the best predictor(s), ambiguities exist in determining the biologically optimal time(s) for data acquisition. Earlier acquisition data, if predictive of grain yield, would allow earlier estimates, decision making and interventions to be made. Previous studies have reported different times of data acquisition over the vegetative growth and reproductive stages–from a short period of time [14, 22] and as little as one flight day [26], up to tens of times throughout the growing season [10, 23, 33]. UAS data collection can be time consuming and expensive, thus, it may not be economic or practical to acquire UAS images throughout the growing season. Previous attempts were made to generate early detectors of yield using satellite images over a large landmass [31, 45, 46], as well as for experimental field [47]. However, none of the aforementioned approaches investigated over numerous genotypes, with different planting times, together. Though an approach using UAS for numerous inbred hybrids of maize was made using Genome to Fields GxE project data” (http://www.genomes2fields.org/, accessed March 9^th^, 2022), the approach treated optimally planted and delayed planted crops separately [31]. Here two differently planted experiments were combined. In each, 25 UAS flights throughout growth were pared down to 12 high quality flights so that biophysical, rather than technical, understanding could be achieved, and early and late planted crops were treated within same model.

The previously referenced temporal models attempt to estimate yield (a cumulative effect) via a series of time series data [8, 10, 23, 24, 30]. Thus, investigating applicability of ΣVIs as predictors would be broadly useful if such models can reduce the number of needed UAS flying dates. Similarly, there is no definitive indication on the best time in the season for data acquisition. To test possibilities of reducing the number of flying days, in this study, the entire growing season (from emergence to harvest) was split into vegetative growth and reproductive stages. The vegetative growth stage was considered from crops emergence up until the crops reached a stable height, and the reproductive stage was considered from the end of vegetative growth stage until the harvest.

The specific objectives of this study were to determine (1) if ΣVIs can be a good alternative to time series VIs and/or CHM data; (2) if the number of UAS data acquisition times/days can be reduced; (3) if any reduced model (with lesser number of predictors) can serve as a robust model; and (4) if there is any biological or physical basis to these results that could be more broadly applicable to UAS in small plot analysis. Physical interpretation of the most important VIs for predicting yield would be beneficial for biologists. Overall, a better understanding of how remote sensing measures can be routinely and easily used across environments and germplasm will allow better predictions for plant breeding and precision agriculture.

## Materials and methods

### Field breeding experiment

The field experiment was conducted near College Station, TX, (30°32’46.3"N 96°26’00.2"W) during the field season of 2019. The field was subdivided into two trials. Each trial contained two replicate blocks of 100 genetically distinct hybrids. Each trial was planted in 13 equal ranges of seven m long along one straight line (tractor row), with 32 such tractor rows, parallel to each other, and separated by 76 cm. The two trials differed from each other in planting dates, management practices and randomization of hybrid position. In one trial, seeds were planted near the optimal planting time (March 21st, 2019) and were properly irrigated (IHOT). In the other trial the seeds were planted later and with less fertilizer to induce stress (April 12th, 2019), these were maintained as dryland (DHOT). The different hybrids were derived from elite but diverse commercial and Texas A&M inbred lines [8]. More details on data acquisition and data processing are available from Adak et al. [8].

### UAS based image collection and analysis

Temporal images were acquired using a DJI Phantom 4 Pro V2.0 UAS, manufactured by DJI (Shenzhen, China). The field of view (FOV) was 84°, focus capacity ranged from f/2.8 to f/11. The UAS was flown at a height of 25m above ground. The camera installed in the UAS captured images in the visible range (red-green-blue, or RGB). A total of 45 ground control points were used for georeferencing the UAS captured images for this and other adjacent trials. Several images were acquired from each field, per flight. All images were orthomosaicked to generate one image per flight per field (AgiSoft, St. Petersburg, Russia). There were 25 days in total where UAS images were taken, spread over both vegetative growth and reproductive stages. However, after initial quality checks, a subset of 12 days were selected for generating the final VIs (six over the growth stage until flowering, and six over the reproductive stage). The VIs were generated from 8-bit RGB images. To estimate VIs while reducing computing times the images were reduced by aggregating several pixels and effects of soil were eliminated. The final selected images were subjected to additional analysis, such as crop height estimation and vegetation indices [8]. The vegetation indices used were Blue green pigment index (BGI) [48], Brightness index (BI) [49], Excessive green (EXG) [50], Excess green minus excess red index (EXGR) [51], Green leaf index (GLI) [52], Modified green-red index (MGVRI) [53], Normalized difference index (NDI) [54], Normalized green-blue difference index (NGBDI) [55], Normalized green-red difference index (NGRDI) [56], Red-green blue index (RGBVI) [53], Visible atmospherically resistant index (VARI) [57], and Vegetative (VEG) [58]. The S1 Table shows the final list of 12 VIs (along with their corresponding equations) used in the modeling. These VIs were used both as time series observations as well as cumulative VIs. CHMs, however, were not converted into cumulative CHMs. As it can be imagined, crop height itself is a cumulative representation of crop growth stages in vertical direction.

Crop heights were extracted from the 3D point cloud from orthomosaicks belonging to each flight date [8]. The point clouds, after filtering for noise, were converted to digital surface models (DSM) and digital terrain models (DTM). CHM was taken as the difference between DSM and DTM [8]. Additionally, maximum of CHMs and VIs were also estimated. While results of using maximum of CHMs and VIs are briefly discussed but have not been reported extensively, as they did not produce any significantly important results different from what we otherwise discuss.

Best Linear Unbiased Predictors (BLUP) were applied to reduce the number of observations to an entry basis per test (200 in total observations per trial) for both manual and remotely sensed measurements. The BLUP estimate predictors were a function of pedigree, range, row, and replication [8]. The final analysis was continued using various machine learning techniques on these BLUPs.

### Crop yield estimation

Year-end yield was measured from combine harvesting each plot in each trial. A regression model was calculated with yield as the dependent variable from function of independent variables of flight, pedigree, management, spatial variation and their relevant interaction terms to determine variance components and repeatability [8]. Crop yield was used as the predictor.

### Machine learning regression analyses

#### General information on machine learning

Machine learning is a combination of model building and cross validation. A portion of existing data was used for training (model building). The rest was used as validation data in a cross-validation approach, iteratively. A k-fold cross-validation technique (k = 10) was used with a repetition of three per complete sets of folds. 1000 iterations of each such set were run for each regression and for each model type used. Machine learning is based upon regression modeling, and the simplest form of regression for continuous datasets is linear regressions (least square estimators), which was tested first for each model. Each iteration was further tested with ridge, lasso, elastic net, and random forest regressions. Models were evaluated based on cross validation accuracy of predicting validation data. Each optimization was tested on ΣVIs and time series CHMs at both vegetative growth and reproductive stages. Accuracies reflect the correlation between the actual and predicted validation data. The algorithm generated 1000 accuracies for each model (from 1000 iterations). The final model accuracy for each regression and for each model type was the median of these 1000 accuracies. Additionally, the algorithm identified the most important predictor(s) for each model for each iteration. Prediction accuracy was important for selecting the best predicting models and the most important variable were used to build such models.

#### Machine learning training data percentage optimization

It was observed that accuracies of cross validation were dependent on data splitting. Thus, to optimize the percentage of data to be used as training data a small experiment was conducted. In this experiment the percentage of training data was increased from 50% to 90% (at intervals of 10%) while simultaneously monitoring the accuracies of prediction for each regression model type (ridge, lasso, elastic net, and random forest). To reduce computation time during this optimization, the number of iterations was reduced for each case from 1000 to 200. This optimized percentage of data was used for the final modeling and gave preliminary indications on the accuracy level of each regression type at their optimized conditions for each of the scenarios. These tests were made on time series of CHM, ΣVI-SUMs, and ΣVI-AUCs, separately, for vegetative growth and reproductive stages. Different combinations of these variables (CHM, ΣVI-SUMs, and ΣVI-AUCs, separately, for vegetative growth and reproductive stages) were tested for the yield predicting models.

#### Re-aligning growth stages from both trials

**Fig 1:**
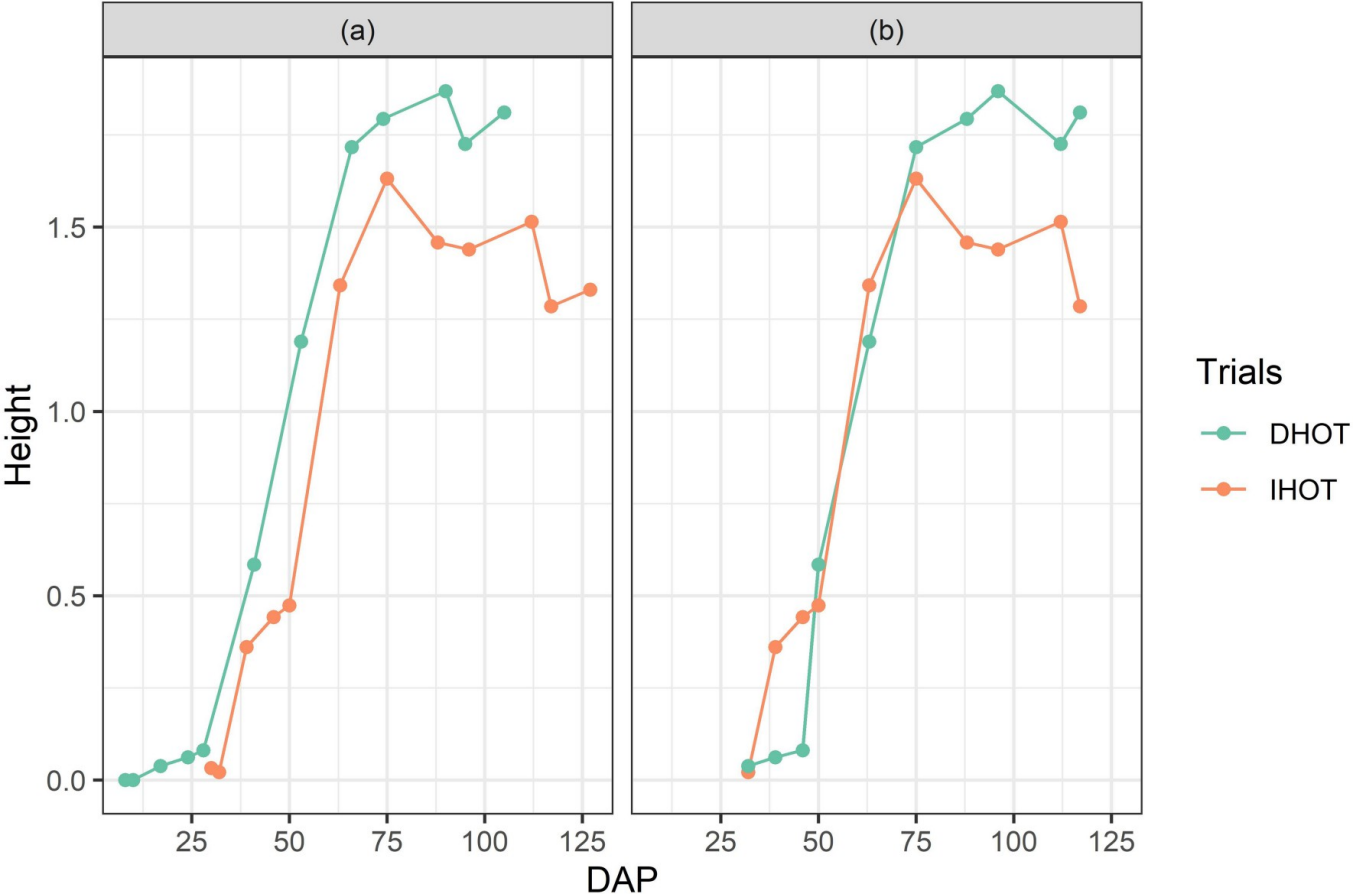
Average crop heights, per trial. (a) Before adjustments, and (b) after adjustments, of UAS observation dates, for re-alignments of crop growth stages at two trials. The Y-axes show the average height per trial, whereas the X-axes show the day after planting (DAP) as of IHOT trial.

Owing to different planting dates, the two trials, IHOT and DHOT, had different growing periods (Fig 1A). For analysis purposes and to maximize statistical power and discovery, trial managements were treated together by stage, rather than by calendar date. DHOT UAS observation dates were shifted by one UAS observation date backward. Simplifying, for the nth UAS observation date (all n^th^ observations, including, yield, CHMs, and all VIs) from DHOT was shifted such that it looked like all those observations belonged to the (n-1)^th^ UAS observation date. All analyses, hereafter, have been referred in terms of day after planting (DAP) of IHOT. After adjustment, growth stages of IHOT and DHOT trials overlapped each other, making them comparable (Fig 1B). Table 1 summarizes initial and final dates of growth stages for both trials.

**Table 1:** Crop growth and reproductive stages of IHOT and DHOT trials in terms of their Days After Planting (DAP), before and after re-alignment of DHOT crops’ growth stage.

	Apr 29^th^, 2019	May 6^th^, 2019	May 10^th^, 2019	May 23^rd^, 2019	Jun 4^th^, 2019	Jun 17^th^, 2019	Jun 25^th^, 2019	Jul 11^th^, 2019	Jul 16^th^, 2019	Jul 26^th^, 2019
**IHOT DAP (Original)**	32	29	46	50	63	75	88	96	112	117
**DHOT DAP (Original)**	10	17	24	28	41	53	66	74	90	95
**DHOT DAP (After re-alignment)**	17	24	28	41	53	66	74	90	95	105

#### Yield predicting models tested

Eight models were generated using different combinations of predictors at different vegetative growth and reproductive stages. Accordingly, models were split into several groups.

Group-I Models: Models in this group used time series data over the entire vegetative growth and reproductive stages. Three models were generated that belonged to this group–(Model-I-1) using time series CHM and time series VIs were both used as predictors, (Model-I-2) using only time series CHM as predictors, and (Model-I-3) using only time series VIs as predictors. The primary purpose of these models was to use as reference models for comparisons with Group-II, Group-III, and Group-IV models that used cumulative predictors.

Group-II Models: Models in this group used cumulative vegetation indices (ΣVIs), and/or time series CHM data, for the vegetative growth stage only. Cumulative VIs (ΣVIs) were calculated two ways–first, as a summation of each individual VI throughout flights (ΣVI-SUMs, s for plural); second, as the area under the curve (ΣVI-AUCs, s for plural). The seven models that belonged to this category were–(Model-II-1) ΣVI-SUMs and time series (not cumulative) CHM of vegetative growth stage as predictors; (Model-II-2) ΣVI-AUCs and time series (not cumulative) CHM as predictors; (Model-II-3) ΣVI-SUMs and CHM of the last day of data acquisition during vegetative growth stage (DAP 63^rd^) as predictors; (Model-II-4) ΣVI-AUCs and CHM of the last day of data acquisition during vegetative growth stage (DAP 63^rd^) as predictors; (Model-II-5). Only ΣVI-SUMs of vegetative growth stage as predictors; (Model-II-6). Only ΣVI-AUCs of vegetative growth stage as predictors. (Model-II-7) Only time series CHM of vegetative growth stage as predictors. Models-II-3 and 4 were generated to test if time series CHM data estimations could be reduced. Here, CHM were replaced by the CHM estimations of the last day of UAS data acquisition during vegetative growth (DAP 63^rd^) stage. These CHM estimations (for DAP 63^rd^) were used as a proxy to maximum crop height during growth vegetative stage.

Group-III Models: Models in this group included all seven models belonging to Group-II, but only for the reproductive stage. Here also, Models-III-3 and 4 were generated to test if time series CHM data estimations could be reduced, by replacing time series CHM estimations with the CHM estimations of the last day of UAS data acquisition during vegetative reproductive stage (DAP 117^th^).

Group-IV Models: The two models belonging to this group was generated as a result from the analyses of the Group-II and Group-III models. The purpose was to test if a reduced number of predictors can generate model(s) with accuracies close to any of the aforementioned models. Model-IV-1 used ΣVI-SUMs of only NRGDVI, NGBDI, and GLI for the vegetative growth stage only, and time series CHM of the vegetative growth stage only. Model-IV-2 used time series NGRDI, NGBDI, GLI, and CHM for DAP 50^th^, DAP 63^rd^ and DAP 75^th^. Both models eliminated the rest of the VIs or their SUMs or AUCs.

#### Additional yield predicting models

In addition to the aforementioned models, additional models were also tested–(Addl.-I) maximum of VIs and maximum of CHMs of the entire growing season (VI_max_s (s for plural) and CHM_max_, respectively) as predictors, and (Addl.-II) only VI_max_s of the entire growing season as predictors.

## Results

### Crop heights and yields from IHOT and DHOT trials

**Fig 2:**
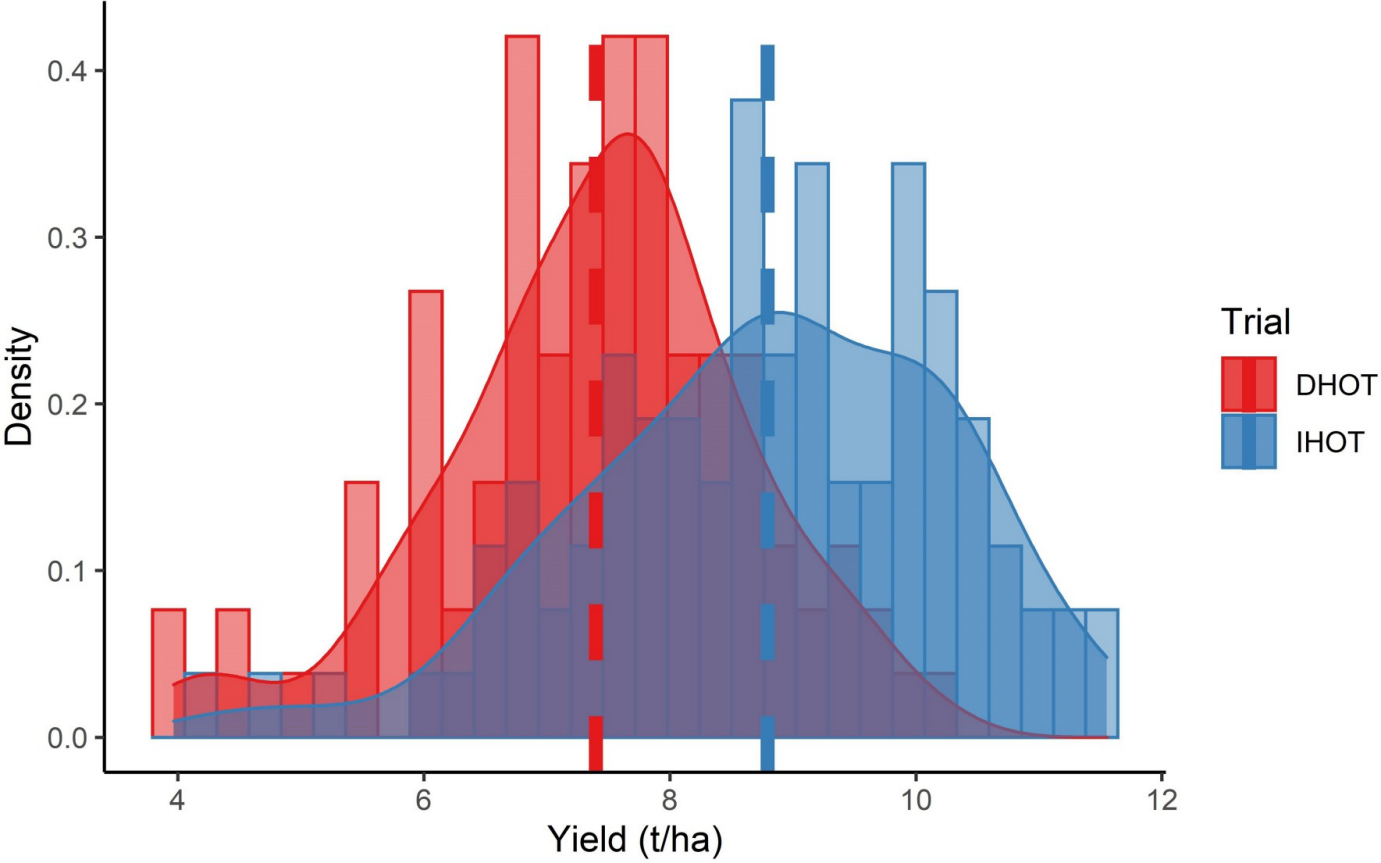
Average crop yield density, as well as histogram, plots, per trial. The Y-axis shows the density of yield, whereas the X-axis shows the grain yield in tons per hectare.

Grain yield from IHOT plots was higher than DHOT. However, IHOT yield values had a wider range than DHOT (Fig 2). The average yield from IHOT was 8.8 t ha-1, whereas that from DHOT was 7.4 t ha-1. The difference between the two means was significant (p<0.05). DHOT likely yielded lower due to more environmental stress, a conjugate effect of moisture stress (drought) and being planted later, which exposed the trial to higher temperatures at critical physiological stages. Despite that crops at IHOT trials yielded higher than crops at DHOT trial (Fig 2), average crop heights at IHOT trial were shorter than the DHOT trial (Fig 1).

### Machine learning training data optimization results

**Fig 3:**
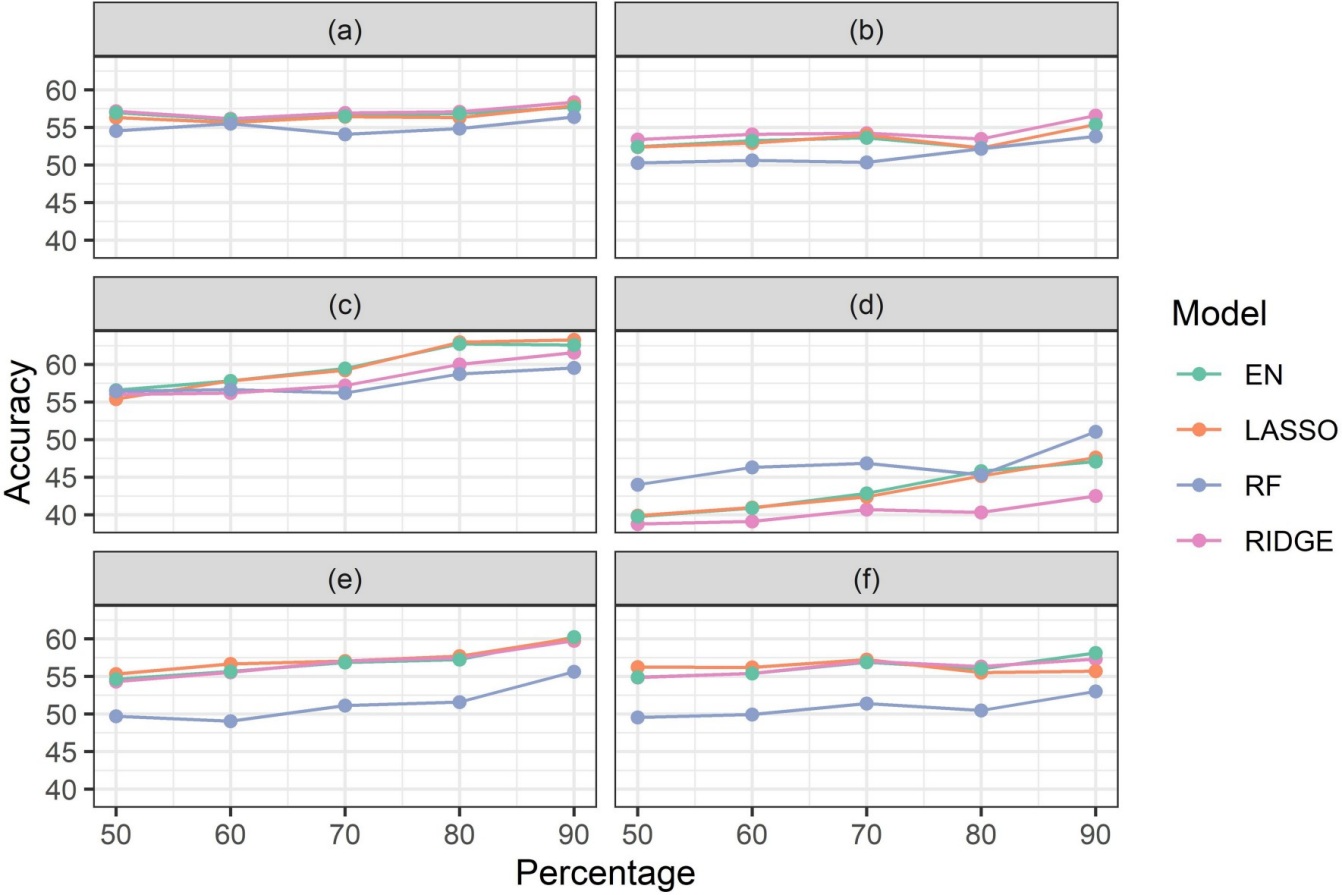
Accuracies generated by different models with different regressions from training data optimization test. The X-axes show “Percentage” of data used as training data and the Y-axes show the corresponding accuracies. For each model results from elastic net (EN), lasso (Lasso), ridge (Ridge), and random forest (RF) have been shown. The models tested were for–(a) time series CHM for vegetative growth stage, (b) time series CHM for reproductive stage, (c) ΣVI-SUMs for vegetative growth stage, (d) ΣVI-SUMs for reproductive stage, (e) ΣVI-AUCs for growth stage, and (f) ΣVI-AUCs for reproductive stage.

Optimization model results were evaluated based on the correlation between actual yield and model predicted yield, in the validation dataset. The algorithm for training data optimization generated columns of up to 200 accuracy values for each regression type of each model, and for each training data percentage tested. The median of these up to 200 accuracy values is reported (against increasing percentages of data used in training (Fig 3). Model accuracies were observed to vary with changing percentages of the training data. These changes also varied from model to model and regression type to regression type. The maximum changes against training data percentages were observed in the model with ΣVI-SUMs for the vegetative growth period (5–8%; Fig 3C), and the minimum changes against changing training data percentage were observed in the model with CHM for vegetative growth stage (about 2%; Fig 3A). For most of the other cases the changes appeared to be around 4–5%. Maximum accuracies were consistently observed at 90% training data percentage (except EN for ΣVI-SUMs, which reduced from 80% training data percentage case by less than 1%) (Fig 3). Thus, 90% data were used as training data percentage for all further models.

### Machine learning prediction results

#### Accuracies of the Group-I full data models

**Fig 4:**
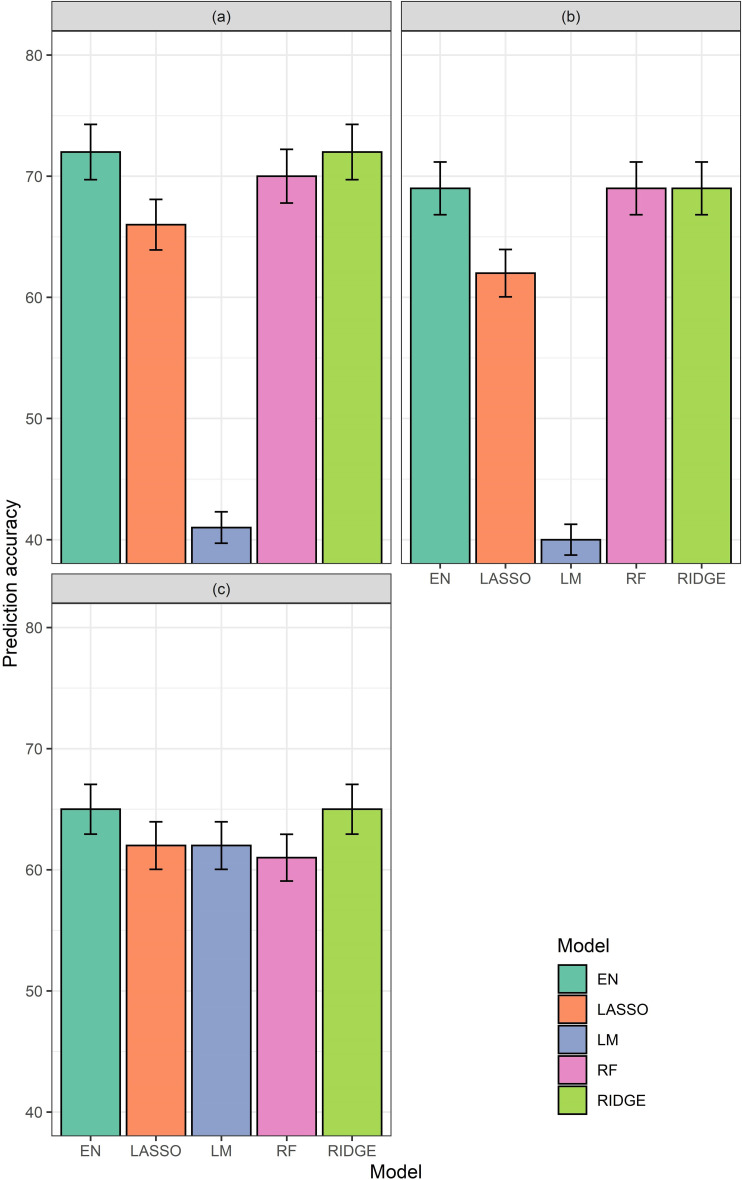
Accuracies generated by different models with different regressions from Group-I models. (a) Model-I-1: Time series VIs and time series CHM for the entire growing season, (b) Model-I-2: Time series VIs only for the entire growing season, and (c) Model-I-3: Time series CHM only for the entire growing season. Accuracies from elastic net (EN), lasso (Lasso), ridge (Ridge), and random forest (RF) models have been shown along the Y-axes.

The Model-I-1 produced maximum accuracies but inconsistent models. The Model-I-1 tested predictabilities of time series of CHM and time series of VIs together in the same model. For example, though EN and RR (~72%) and RF (~70%) produced high accuracies, LM produced accuracies less than 45%. Again, for Model-I-3, which tested the predictabilities of time series CHM only, showed similar inconsistent models. Though, EN, RR, and RF produced accuracies of about 68%, LM produced accuracy even less than 40%. Only when using time series VIs as the only predictors, produced accuracies between 60–65% for all regression type (Model-I-2) (Fig 4).

#### Accuracies of the Group-II vegetative models

**Fig 5:**
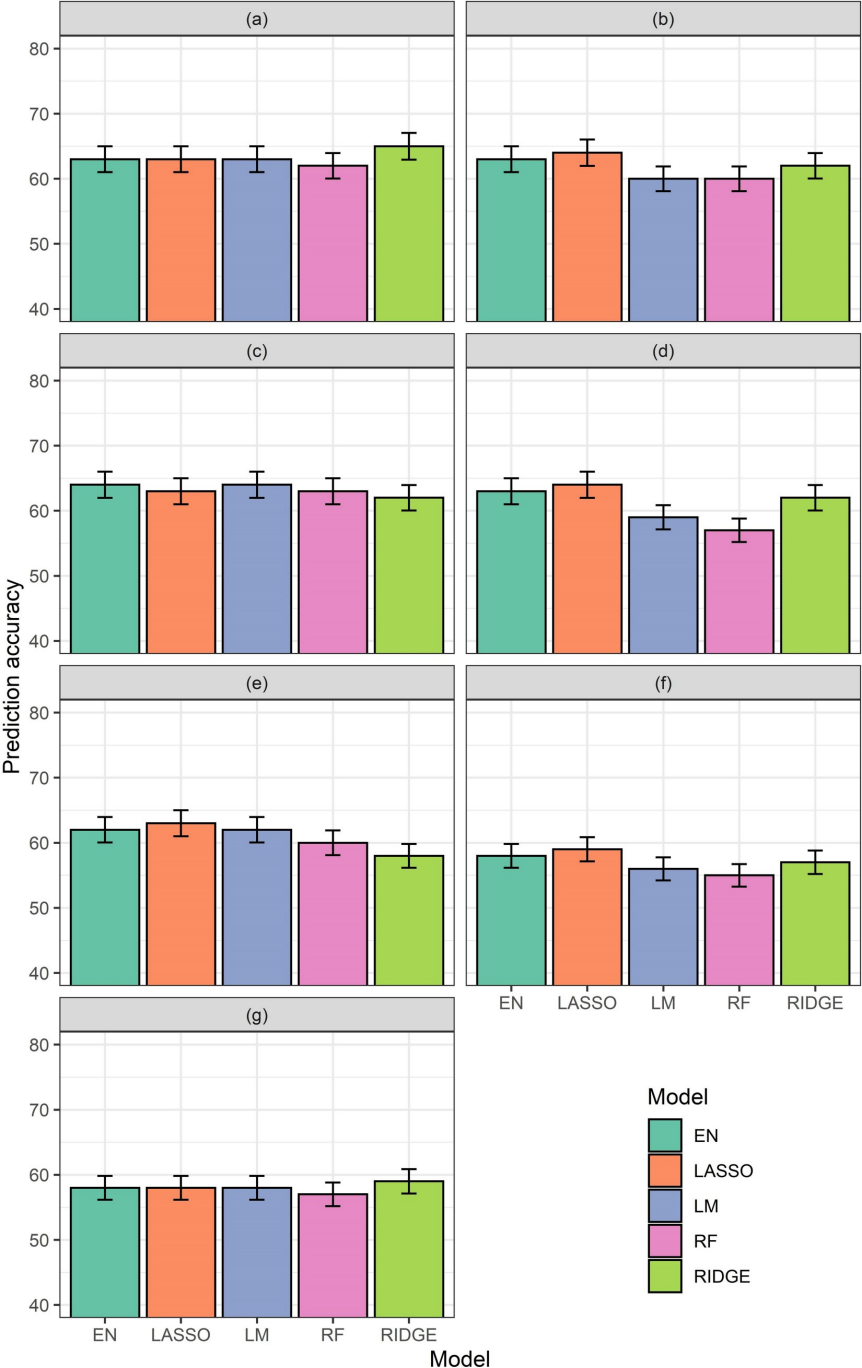
Accuracies generated by different models with different regressions from Group-II models, for vegetative growth stage. (a) Model-II-1: ΣVI-SUMs and time series CHM for vegetative growth stage, (b) Model-II-2: ΣVI-AUCs and time series CHM for vegetative growth stage, (c) Model-II-3: ΣVI-SUMs and CHM of DAP 63^rd^ for vegetative growth stage, (d) Model-II-4: ΣVI-AUCs and CHM of DAP 63^rd^ for vegetative growth stage, (e) Model-II-5: ΣVI-SUMs for vegetative growth stage, (f) Model-II-6: ΣVI-AUCs for vegetative growth stage, and (g) Model-II-7: Time series of CHM for growth stage. Accuracies from elastic net (EN), lasso (Lasso), ridge (Ridge), and random forest (RF) models have been shown along the Y-axes.

Among Model-II-1, which tested ΣVI-SUMs and time series CHM as predictors, and Model-II-3, which tested ΣVI-SUMs and CHM of DAP 63^rd^ as predictors, produced similar consistent models. All models in these two groups produced accuracies between 60–65%. However, when in the aforementioned models ΣVI-SUMs are replaced by ΣVI-AUCs, EN, LR, and RR produced accuracies 60–65%, but LM and RF produced accuracies between 55–60%. ΣVI-SUMs alone (Model-II-5), were better, except for RR, predictors than ΣVI-AUCs alone, or time series CHM, alone (Fig 5).

#### Accuracies of the Group-III reproductive models

**Fig 6:**
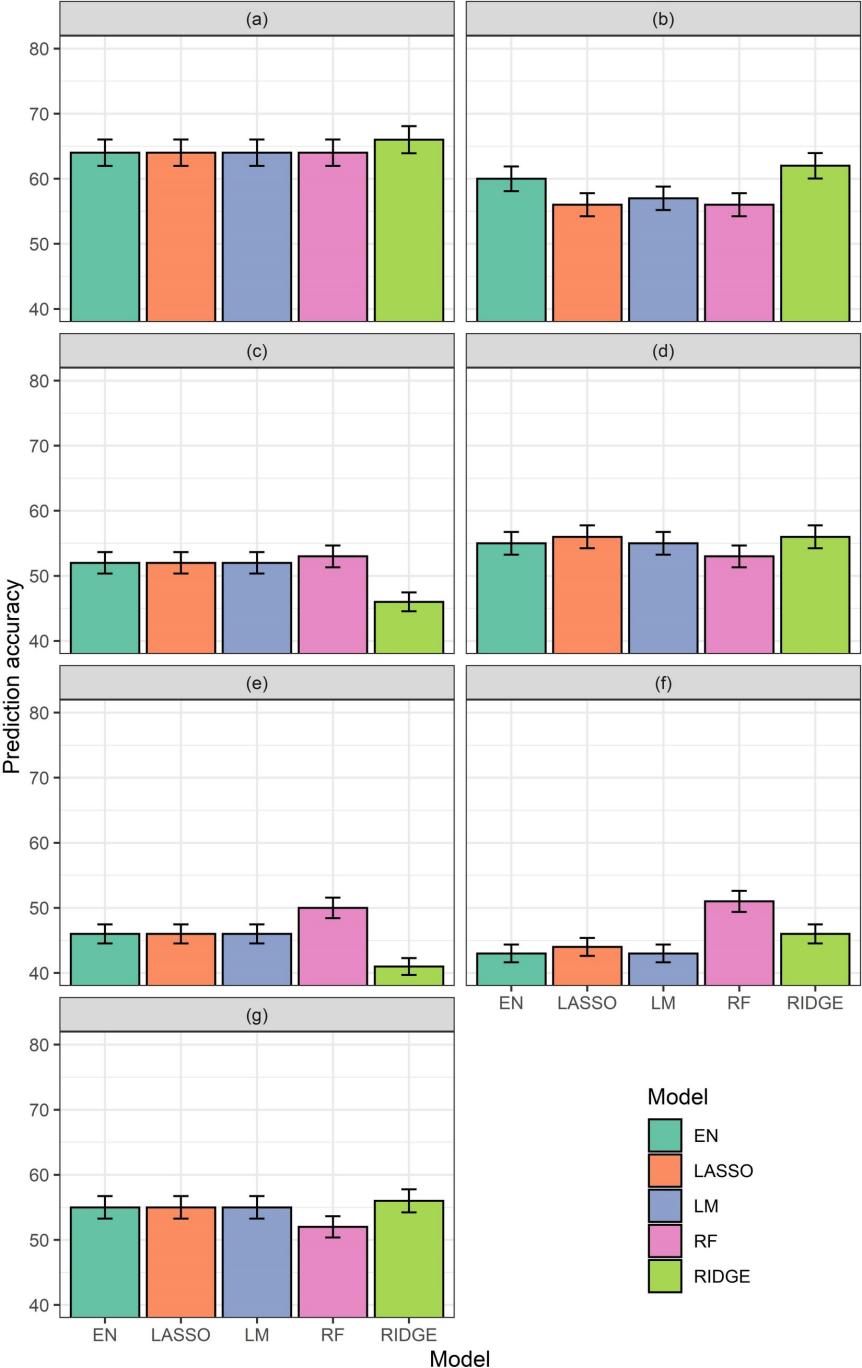
Accuracies generated by different models with different regressions from Group-III models, for vegetative reproductive stage. (a) Model-III-1: ΣVI-SUMs and time series CHM for vegetative reproductive stage, (b) Model-III-2: ΣVI-AUCs and time series CHM for vegetative reproductive stage, (c) Model-III-3: ΣVI-SUMs and CHM of DAP 117^th^ for vegetative reproductive stage, (d) Model-III-4: ΣVI-AUCs and CHM of DAP 117^th^ for vegetative reproductive stage, (e) Model-III-5: ΣVI-SUMs for vegetative reproductive stage, (f) Model-III-6: ΣVI-AUCs for vegetative reproductive stage, and (g) Model-III-7: Time series of CHM for reproductive stage. Accuracies from elastic net (EN), lasso (Lasso), ridge (Ridge), and random forest (RF) models have been shown along the Y-axes.

Except for Model-III-1, 4, and 7, all model groups produced poor results. Among these three groups, only Model-III-1, which tested predictabilities of ΣVI-SUMs and time series CHM, produced accuracies between 60–65%. On the contrary, models with ΣVI-AUC and CHM of DAP 117^th^, and time series of CHM only produced accuracies between 50–55%. All other models in this group produced poor accuracies and inconsistent results (Fig 6).

#### Accuracies of the Group-IV refined models

**Fig 7:**
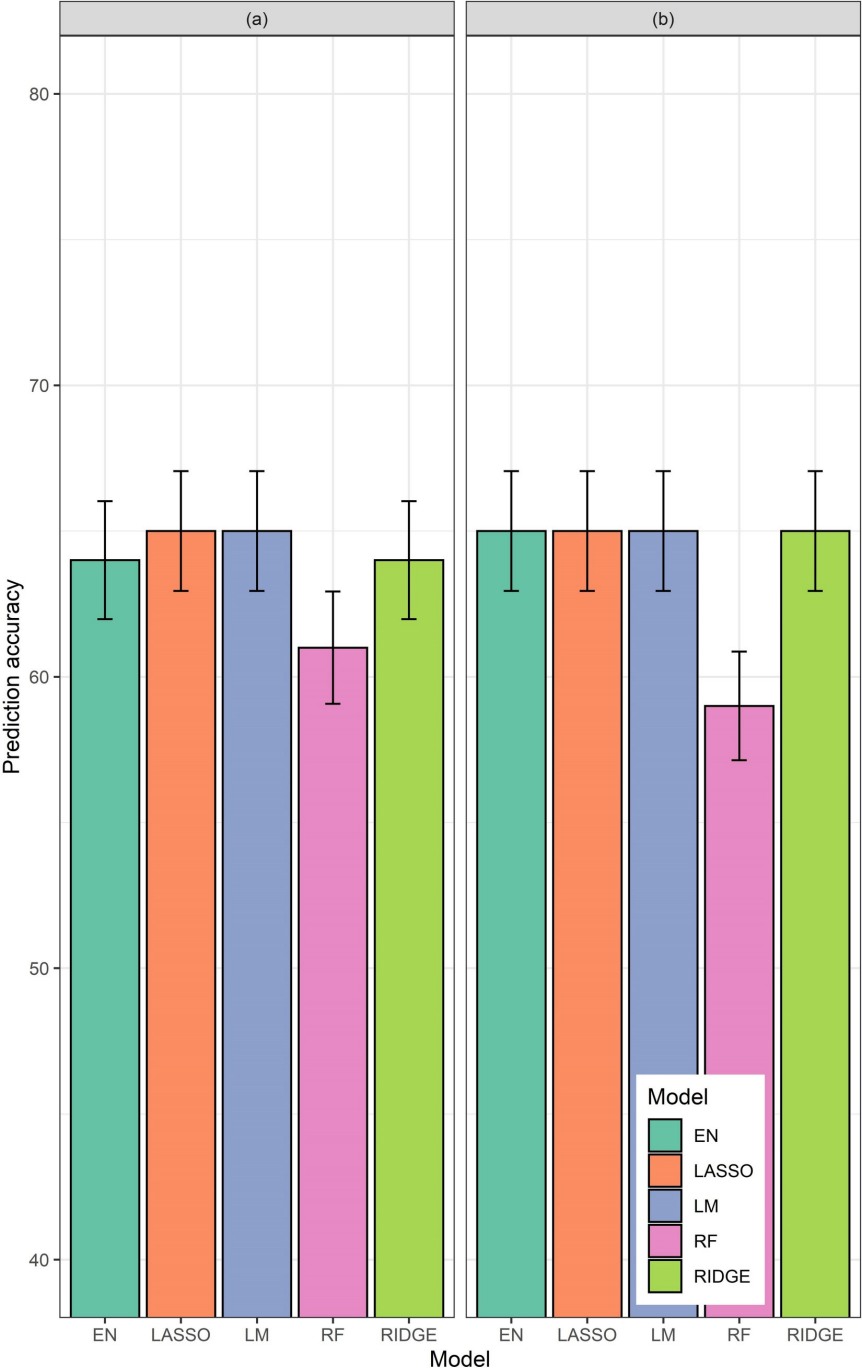
Accuracies generated by different models from Group-IV. (a) Model-IV-1: using ΣVI-SUMs of NGRDI, NGBDI, and GLI only, and time series of CHM, for vegetative growth stage, as predictors, (b) Model-IV-2: time series NGRDI, NGBDI, GLI, and CHM for DAP 50^th^, DAP 63^rd^ and DAP 75^th^. Accuracies from elastic net (EN), lasso (Lasso), ridge (Ridge), and random forest (RF) models have been shown along the Y-axes.

Based on the results of Group I, II and III models, additional models were tested. Model-IV-1 using ΣVI-SUMs of NGRDI, NGBDI, and GLI only, and time series of CHM, for vegetative growth stage, as predictors produced 65% accuracies for LR and LM, and marginally below 65% for EN and RR (Fig 7A). Model-IV-2 with time series of NGRDI, NGBDI, GLI, and CHM, for DAPs 50th, 63rd, and 75th only, produced accuracies of 65%, except RF (Fig 7B).

#### Accuracies of the additional model

The additional models did not produce any significant differences, from aforementioned sections. For example, when ΣVI-SUMs and cumulative CHM, both for entire growing season were used as predictors (Addl.-I), except for RF, all regression types produced 65–66% accuracies. The same regression types produced 68–69% accuracies when VI_max_s and CHM_max_ for entire growing season were predictors (Addl.-II). Complete absence of CHM (Addl.-III) did not make any significant changes to accuracies, except for RF. Models with ΣVI-SUMs for growth stage only and CHM_max_ produced consistent accuracies of 67% by all regression models.

#### Importance of variables

For Group I models, each regression of each different model type identified the most important variables in the modeling. Some consistencies were observed among different models and regression types. For example, for both Group-I Model 1 and Model 3, EN, Lasso, and Ridge regressions predicted NGRDI (but for different dates of acquisitions) as the best predictor. RF, however, identified VARI of DAP 50^th^ as the best predictor for Model 1. Group-II Model 2 identified CHM acquired during vegetative reproductive stage as the best predictor (Table 2).

**Table 2:** Results of Group-I models. The results include results from regressions for each model, and most important variables for each model for each regression type, and accuracies of each model for each regression type.

Model Type	Regression type	Most important variable	Accuracy (%)
**Group-I model 1 of 3:** Time series CHM and all VIs over vegetative growth and reproductive stages as predictors	Elastic net	NGRDI of DAP 63	72 (±2.28)
	Lasso	NGRDI of DAP 39	66 (±2.09)
	Ridge	NGRDI of DAP 88	72 (±2.28)
	Random Forest	VARI of DAP 50	70 (±2.21)
**Group-I model 2 of 3:** Time series CHM over vegetative growth and reproductive stages as predictors	Elastic net	DAP 75	65 (±2.18)
	Lasso	DAP 46	62 (±1.96)
	Ridge	DAP 88	65 (±2.18)
	Random Forest	DAP 75	61 (±2.18)
**Group-I model 3 of 3:** Time series VIs over vegetative growth and reproductive stages as predictors	Elastic net	NGRDI of DAP 50	69 (±2.06)
	Lasso	NGRDI of DAP 39	62 (±1.96)
	Ridge	NGRDI of DAP 63	69 (±2.06)
	Random Forest	VARI of DAP 50	69 (±1.93)

Among Group-II models, again EN, Lasso, and Ridge regressions identified NGRDI as best predictor for Models 2, 4, and 6. Lasso regressions alone again identified NGRDI as best predictor in Model 5. EN and Lasso regressions of Model 1 and EN and Ridge regressions of Model 5 identified GLI as best predictor. CHM data acquired during 50^th^ DAP was the best predictors for all the regressions of Model 1. Again, Ridge and RF predicted CHM acquired near the end of vegetative growth stage of Model 1 and Model 3 (Table 3).

**Table 3:** Results of Group-II models. The results include results from regression types for each model (column 2), and both most important variables for each model for each regression type (column 3), and accuracies of each model for each regression type (column 4).

Model Type	Regression type	Most important variable	Accuracy (%)
**Group-II model 1 of 7:** Time series CHM and ΣVI-SUMs over vegetative growth stage as predictors	Elastic net	GLI	63 (±1.99)
	Lasso	GLI	63 (±1.99)
	Ridge	CHM of DAP 63	65 (±2.06)
	Random Forest	CHM of DAP 50	62 (±1.96)
**Group-II model 2 of 7:** Time series CHM and ΣVI-AUCs over vegetative growth stage as predictors	Elastic net	NGRDI	63 (±1.99)
	Lasso	NGRDI	64 (±2.02)
	Ridge	NGRDI	62 (±1.96)
	Random Forest	CHM of DAP 63	60 (±1.90)
**Group-II model 3 of 7:** CHM of DOY 63 and ΣVI-SUMs over vegetative growth stage as predictors	Elastic net	NGBDI	64 (±2.02)
	Lasso	NGBDI	63 (±1.99)
	Ridge	CHM of DAP 63	62 (±1.96)
	Random Forest	CHM of DAP 63	63 (±1.99)
**Group-II model 4 of 7:** CHM of DOY 63 and ΣVI-AUCs over vegetative growth stage as predictors	Elastic net	NGRDI	63 (±1.99)
	Lasso	NGRDI	64 (±2.02)
	Ridge	NGRDI	62 (±1.96)
	Random Forest	CHM of DAP 63	57 (±1.80)
**Group-II model 5 of 7:** ΣVI-SUMs over vegetative growth stage as predictors	Elastic net	GLI	62 (±1.96)
	Lasso	NGRDI	63 (±1.99)
	Ridge	GLI	58 (±1.83)
	Random Forest	NDI	60 (±1.90)
**Group-II model 6 of 7:** Type-II ΣVIs over vegetative growth stage as predictors	Elastic net	NGRDI	58 (±1.83)
	Lasso	NGRDI	59 (±1.87)
	Ridge	NGRDI	57 (±1.80)
	Random Forest	NGRDI	55 (±1.74)
**Group-II model 7 of 7:** Time series CHM vegetative growth stage as predictors	Elastic net	DAP 50	58 (±1.83)
	Lasso	DAP 50	58 (±1.83)
	Ridge	DAP 50	59 (±1.87)
	Random Forest	DAP 50	57 (±1.80)

In the Group-III models NGRDI was also identified as best predictors by EN in Model 1 and RF in Model 6. GLI was a common VI predictor for Group-III models, as indicated EN, Lasso, and Ridge of Model 1, and EN and Lasso of Model 4 and 7. RF identified BI to be important predictor for Models 2 and 4. Another very important predictor was CHM of DAP 75^th^, as consistently identified by all regressors of Model 7, and EN and Ridge regressions of Model 1 (Table 4).

**Table 4:** Results of Group-III models. The results include results from regression types for each model (column 2), and both most important variables for each model for each regression type (column 3), and accuracies of each model for each regression type (column 4).

Model Type	Regression type	Most important variable	Accuracy (%)
**Group-III model 1 of 7:** Time series CHM and Type-I ΣVIs over vegetative reproductive stage as predictors	Elastic net	CHM of DAP 75	64 (±2.02)
	Lasso	NGBDI	64 (±2.02)
	Ridge	CHM of DAP 75	66 (±2.09)
	Random Forest	NDI	64 (±2.02)
**Group-III model 2 of 7:** Time series CHM and Type-II ΣVIs over vegetative reproductive stage as predictors	Elastic net	NGRDI	60 (±1.90)
	Lasso	NGBDI	56 (±1.77)
	Ridge	NGBDI	62 (±1.96)
	Random Forest	BI	56 (±1.77)
**Group-III model 3 of 7:** CHM of DOY 117 and Type-I ΣVIs over vegetative reproductive stage as predictors	Elastic net	GLI	52 (±1.64)
	Lasso	GLI	52 (±1.64)
	Ridge	GLI	46 (±1.45)
	Random Forest	CHM of DAY 117	53 (±1.68)
**Group-III model 4 of 7:** CHM of DOY 117 and Type-II ΣVIs over vegetative reproductive stage as predictors	Elastic net	NGBDI	55 (±1.74)
	Lasso	BGI	56 (±1.77)
	Ridge	NGBDI	56 (±1.77)
	Random Forest	BI	53 (±1.68)
**Group-III model 5 of 7:** Type-I ΣVIs over vegetative reproductive stage as predictors	Elastic net	GLI	46 (±1.45)
	Lasso	GLI	46 (±1.45)
	Ridge	NGRDI	41 (±1.30)
	Random Forest	VARI	50 (±1.58)
**Group-III model 6 of 7:** Type-II ΣVIs over vegetative reproductive stage as predictors	Elastic net	GLI	43 (±1.36)
	Lasso	GLI	44 (±1.39)
	Ridge	NGRDI	46 (±1.45)
	Random Forest	NGRDI	51 (±1.61)
**Group-III model 7 of 7:** Time series CHM vegetative reproductive stage as predictors	Elastic net	DAP 75	55 (±1.74)
	Lasso	DAP 75	55 (±1.74)
	Ridge	DAP 75	56 (±1.77)
	Random Forest	DAP 75	52 (±1.64)

## Discussion

### Variations in crop height

Due to technical issues associated with UAS imaging (flowering, high wind speeds, etc.) UAS measured crop height fluctuated slightly over time, especially during the taller reproductive stage where wind moved the plants more. The crop height in the IHOT trial remained taller than in the DHOT trial during the entire vegetative growth stage as the IHOT trial was planted earlier. By reproductive stage, however, plants in the DHOT trial were taller (Fig 2A). DHOT plots were possibly taller during the reproductive phase because of exposure to the longer days during vegetative growth stage; this is frequently seen in the Central Texas environment. DHOT yielded less grain (1.4 t ha-1), so a higher crop height did not necessarily mean higher yield when combining both trials. Thus, crop height alone may be a reliable predictor of yield within each trial, but not between trials, especially when environmental stress is involved.

### Importance of VI based models

Historically CHM [10, 13–16] and VIs [21–26] have been used in predicting yield from segregating trials and agronomic experiments of single environments. However, this study demonstrates how CHM alone may not be as good of a predictor of yield between trials. Despite having greater average vegetative reproductive stage heights (CHM), DHOT crops yielded less than IHOT crops (Figs 2 and 3). Comparisons between three Group-I models using all data suggest, when used alone as predictors, VIs were better predictors of yield than CHM alone, but combining both was best. The best regression model of Group-I, Models-I-1 produced over 70% accuracy, whereas, when only CHMs were predictors (Model-I-2), the prediction accuracy of the best regression models dropped to around 65%. It can be concluded that within the same environment CHM may be a good predictor, but when different environments (e.g., irrigated and dryland, for this case) are combined VIs alone can be more predictive. More generally, for yield estimation, representations of both structural and morphological traits have the greatest predictive power, and thus CHM and VIs together produced best models.

### Properties of VI based models

The primary disadvantage of Group-I models was the resources needed to acquire and process UAS data throughout the growing season (emergence to harvest). Group-II and Group-III models tested the possibilities of reducing data acquisition, confining flights within either vegetative growth or reproductive stage. Group-II vegetative growth models would be preferred because they would allow decisions to be made earlier in the season. The overall results suggest vegetative growth stage models were indeed better predictors of grain yield than reproductive stage models. When applied with CHM, there was no noticeable difference between ΣVI-SUMs and ΣVI-AUCS based models when using EN, LR or RR, which all had accuracies around 60%-65%. Only, when used with CHM, for LM and RF, were ΣVI-SUMs models better predictors than ΣVI-AUCs used with CHM. When only ΣVIs were used as predictors, ΣVI-SUMs based models were consistently better (60%-65%) predictors (except LM) than ΣVI-AUCs based models (55%-60%).

Overall, these results show a substantial prediction advantage to summing VI’s than to taking the area under their curves. In simple summation, each time point acts independently of the previous timepoints; for the area under the curve, the value of the adjacent time points are important to calculating the final value. AUC thus buffers extreme timepoints to be less influential that they would be in summation, which appears to reduce prediction. It is important to note that for the predictions made here, the comparative values between genotypes are being evaluated in the same environments and flights. Therefore, if a single UAS flight in a single environment has extreme VIs across all plots it becomes a constant and will not affect comparisons between genotypes within that environment; however, it is likely that an extreme flight could have an outsize effect on comparisons between environments. We were highly selective in the flights used (12 of 25 days possible) based on having the highest quality data; therefore, the advantage of ΣVI-SUMs suggests that biologically, VIs are temporally independent in their ability to predict yield, with the highest timepoints having the most impact on yield. However, it seems likely that with less technical quality control, such as poorly cleaned data, or environments with sparse flights AUC could be able to better smooth over single flight issues. For these reasons it is worthwhile to continue to calculate and evaluate both the ΣVI-SUMs and the ΣVI-AUCs, which is also easy to do, similar to how multiple machine learning models can be calculated.

As first observed in Group-I, both Group-II and Group-III models also depicted that time series CHM, alone, were poorer predictors of yield than that when time series CHM were used in addition to ΣVIs. Overall, these analyses suggest that if data is collected frequently over the entire season, time series CHM with time series VIs were better though inconsistent predictors. This is logical in that there are more predictor and observations to include to capture differences. In contrast, cumulative single predictors were more robust to differences in UAS flight dates and plant growth stages across environments when data sets needed to be combined. These resulted in only a small sacrifice (2–3%) for yield prediction ΣVI-SUMs based models when combined with time series CHM during the vegetative growth stage only, which produced a more robust model. Such yield estimation throughout the vegetative growth stage could be useful to predict yield in near real-time, as well as to take corrective actions to prevent yield loss, such as in precision input management or integrated pest management (IPM). Otherwise, models that require end of season measurements are primarily useful for information purposes and not for crop management to retain high yields [59]. Thus, this procedure is potentially capable of reducing the UAS data collection to half, confining within vegetative growth stage only, which could also speed the breeding cycle and improve agronomic management. Models based on ΣVI-SUMs with time series CHM throughout the vegetative growth stage, or with CHM acquired on the last day of UAS data acquisition during growth stage (DAP 63rd), did not make any significant difference.

### Best predictors

#### Observed most important times for data acquisitions

Considering all important predictors, it was evident that the most important VIs were NGRDI, NGBDI, and GLI. The most important DAPs for data acquisition were 75^th^, 63r^d^, and 50^th^. Among these, the 75^th^ DAP was the first day of the reproductive growth stage, according to divisions assumed for this study. The 63^rd^, and 50^th^ DAPs however, belong to vegetative growth stage. Here, the important variables selected by machine learning algorithm were all before blister stage, shortly after pollination when the maize kernels are just forming [60]. It is unknown if the importance of this late vegetative to early reproductive period was unique to this studies germplasm and environments, or if it has greater general applicability. Many previous physiological studies in maize have focused on the flowering and grain-filling stages as the important determinants for yield, especially under drought stress [61, 62]. There are three primary possibilities for why our results differ from previous studies. First, the germplasm or Texas environments used in this study could by unique, this will only be discovered through additional investigations and a body of knowledge being built. Second, in the present study, correlations with yield are solely based on segregation between the elite hybrid varieties evaluated. This means that any growth or vegetative stage that genotypes are restricted for in physiological variation for will not be observed as important to determining grain yield. It is indeed possible that breeders have selected and fixed most of the important variation in the reproductive stage; the present studies novel UAS monitoring of the growth stage is where selection could still work and where the most gains could be made. Finally, it is conceivable that the UAS measurements better allow detection of certain types of variation while classical physiological measurements allow better detection of others, as they are not measuring the same things. In this final case these tools could complement each other well.

#### Nature of the equations of the most important vegetation indices

It is evident from Tables 2–4, that out of twelve (12) VIs tested, NGRDI, GLI, and NGBDI had the highest predictability for maize grain yield. Thus, it was useful to understand if any connection existed between these three VIs. The equations for these VIs are (S1 Table for reference):

$$NGRDI=\frac{G-B}{G+B}$$ (1)

$$NGBDI=\frac{G-R}{G+R}$$ (2)

$$GLI=\frac{2G-B-R}{2G+B+R}=\frac{2G-(B+R)}{2G+(B+R)}$$ (3)

where G, B, and R (in the above equations) represent reflectance from green, blue, and red lights.

From healthy green plants, among G, R, and B, green light reflected most, and red and blue get absorbed [27]. Thus, from this concept and assuming B+R≈2R or 2B, the above equations can be given a general form:

$$VI=\frac{\left(High\;reflectance-Low\;reflectance\right)}{\left(High\;reflectance+Low\;reflectance\right)}$$ (4)

Thus, no matter which VIs were investigated to estimate yield, this specific VI type (normalized difference form) was most useful in detecting meaningful variation for grain yield in this study. This result demonstrates that when physical laws behind observational data are obscured, statistical methods can reveal the physical laws. So, for future research, instead of investigating which VI is a best predictor or investigating all types of vegetation, it may be useful to narrow down to those with the type of Eq 4, and investigate the nature of temporal variations of VIs of the aforementioned specific type.

### Best algorithms

Though separate regression models were investigated for the entire season, models developed for vegetative growth stage alone (Tables 2–4), elastic net, or lasso, or ridge regressions were the most accurate. Only in Group-III, Models-III-3, 5, and 6, random forest was the best predictor of yield. In fact, for several occasions random forest predicted yield with significantly lower accuracies than other regression types. Random forest modeling functions are based on classification models, whereas ridge, lasso, and elastic net regressions are modifications of linear models. Thus, these results support previous research reporting the existence of linear relationship between VIs and maize yield [22, 29]. Random forest might be useful for situations when there is difference in the basal level of yield between two groups, which was not the case for this study.

The Model-I-1 of Group-I models produced the maximum accuracy among all the models in all the Groups-I, II, and III, tested. However, the model results were inconsistent across regression approaches. For example, despite EN and RR of Model 1 of Group-I produced maximum accuracy (> 70%), in the same model LM couldn’t exceed 45% accuracy level. This is likely related to variable redundancy present in Model 1 of Group-I, which cumulative models minimize. Thus, Group-IV models were generated, analyzing results of all the models from Group-I to III, by picking up best predictors and best times for measuring CHM. Two models were generated. Since, a model with ΣVI-SUMs and time series CHM in Group-II was good predictor, Model-IV-1 was made with ΣVIs of NGRDI, NGBDI, and, GLI, and time series CHM for the vegetative growth stage only. Model-IV-2 was made with the time series best predictor VIs and time series CHM, and NGRDI, NGBDI, and, GLI, for DAP 50^th^, 63^rd^, and 75^th^ DAP only. The later dates, as mentioned above, matched with blister stage of maize kernel development. Model-IV-2 was the better and consistent predictor (all regressions produced accuracies above 65%), this gave an indication that serious investigation might be necessary between DAP 50^th^ and 75^th^. This time frame also correlates with the plant’s maturity stage (transition from vegetative growth to transition stage), the time when dry matter accumulation reached a maximum [39].

### Additional models

A few tested additional models were not presented in any details because of–(i) the questionable reproducibility of the models and (ii) the models failed to improve the prediction accuracies significantly beyond other models tested. For example, VI_max_s and CHM_max_ were calculated from the maximums of the data used in the model. Calculating actual seasonal maximum may be an expensive and challenging task, requiring condensed data acquisition near-about the times of seasonal maxima. Additionally, for CHM_max_, CHM itself is a representation of cumulative effect of accumulated biomass over the season [63]. So, cumulative CHM might be an overly used representation of crop growths. Thus, overall, the models in this group were not recommended any further.

## Conclusion

This investigation again demonstrated that UAS measured RGB based VIs are useful and can be important for estimating grain yield of maize in segregating populations across environments, when different environmental stresses are involved. Even in elite breeding hybrids, VIs are generally more sensitive to the impact of environmental stresses than crop height. Thus, though crop height may be a good predictor under specific environments for maize, VIs are indispensable under stressed conditions and across environments. Models developed between DAP 50^th^ and 75^th^ (flowering to blister stage) produced accuracies as close to the model with data from the entire season. One major research gap identified in this study was concrete correlations between physical laws/models and regression-based models. We found the most important VIs, as suggested by regression models (with a few simple assumptions) are of the normalized difference type. This research recommends normalized difference type of VIs should primarily be used to estimate yield, and the linear relationships should be expected between normalized difference VI and yield. This may be an indication that when physical laws behind some observational data are obscured, proper use of statistical methods may lead to discovering the physical laws.

This research will lead to several new research directions in the future. For example, in this study reflectance by Near Infra-red (NIR) or Red-shift were not included. It would be important to investigate if presence of NIR and/or Red-shift also lead to the similar results. So, in the future research may focus on—(i) utilizing NIR and Red-shift; (ii) rearranging the flying dates so that cumulative VIs, and maximum of VIs can be estimated testing normalized difference type VIs to see which ones are most useful; and (iii) focusing on developing physiological modeling.

## Supporting information

S1 Table List of the vegetation indices generated (adapted from Adak et al. (2021)). (DOCX)
