# Supplementary material for: Cumulative temporal vegetation indices from unoccupied aerial systems allow maize (Zea mays L.) hybrid yield to be estimated across environments with fewer flights
Source: PLoS One. 2023 Jan 26;18(1):e0277804. doi: 10.1371/journal.pone.0277804 (PMC9879521; doi:10.1371/journal.pone.0277804)
Supplement: S1 Table — (DOCX) [file pone.0277804.s001.docx]

**Table 1S. List of the vegetation indices generated** (adapted from Adak et al. (2021)).

| **Vegetation Indices (VI)** | **Equations** | **References** |
| --- | --- | --- |
| Blue green pigment index (BGI) | $\frac{B}{G}$ | Zarco-Tejada et al. (2005) |
| Brightness index (BI) | $sqrt(\frac{R^{2}+G^{2}+B^{2}}{3})$ | Richardson and Wiegand (1977) |
| Excessive green (EXG) | $(2*G)-R-B$ | Woebbecke et al. (1995) |
| Excess green minus excess red index (EXGR) | $(3*G)-(2.4*R)-B$ | Meyer and Neto (2008) |
| Green leaf index (GLI) | $\frac{2*G-R-B}{2*G+R+B}$ | Louhaichi et al. (2008) |
| Modified green-red index (MGVRI) | $\frac{G^{2}-R^{2}}{G^{2}+R^{2}}$ | Bendig et al. (2015) |
| Normalized difference index (NDI) | $128*(\left( \frac{G-R}{G+R} \right)+1)$ | Hamuda et al. (2016) |
| Normalized green-blue difference index (NGBDI) | $\frac{G-B}{G+B}$ | Hunt et al. (2016) |
| Normalized green-red difference index (NGRDI) | $\frac{G-R}{G+R}$ | Tucker and Red (1979) |
| Red-green blue index (RGBVI) | $\frac{G^{2}-R*B}{G^{2}+R*B}$ | Bendig et al. (2015) |
| Visible atmospherically resistant index (VARI) | $\frac{G-R}{G+R-B}$ | Gitelson et al. (2002) |
| Vegetative (VEG) | $\frac{G}{R^{0.667}*B^{0.334}}$ | Hague et al. (2006) |

**References**

Adak, A., Murray, S.C., Bozinovic, S., Lindsey, R., Nakasagga, S., Chatterjee, S., Anderson, S.L., II, & Wilde, S. (2021). Temporal Vegetation Indices and Plant Height from Remotely Sensed Imagery Can Predict Grain Yield and Flowering Time Breeding Value in Maize via Machine Learning Regression. REMOTE SENSING, 13, 2141

Gitelson, A.A., Kaufman, Y.J., Stark, R., Rundquist, D. (2002). Novel algorithms for remote estimation of vegetation fraction. *Remote Sens. Environ.*, *80*, 76–87.

Hague, T., Tillett, N.D., Wheeler, H. (2006). Automated crop and weed monitoring in widely spaced cereals. *Precis. Agric.*, *7*, 21–32.

Hamuda, E., Glavin, M., Jones, E. (2016). A survey of image processing techniques for plant extraction and segmentation in the field. *Comput. Electron. Agric.*, *125*, 184–199.

Hunt, E.R., Jr., Cavigelli, M., Daughtry, C.S.T., McMurtrey, J.E., III, Walthall, C.L. (2005). Evaluation of digital photography from model aircraft for remote sensing of crop biomass and nitrogen status. *Prec. Agric.*, *6*, 359–378.

Louhaichi, M., Borman, M., Johnson, D. (2001). Spatially located platform and aerial photography for documentation of grazing impacts on wheat. *Geocarto Int.*, *16*, 65–70.

Meyer, G.E., Neto, J.C. Verification of color vegetation indices for automated crop imaging applications. (2008). *Comput. Electron. Agric.*, *63*, 282–293.

Richardson, A.J., Wiegand, C.L. Distinguishing vegetation from soil background information. (1977). *Photogramm. Eng. Remote Sens.*, *43*, 1541–1552.

Tucker, C.J. (1979). Red and photographic infrared linear combinations for monitoring vegetation. *Remote Sens. Environ.*, *8*, 127–150.

Woebbecke, D.M., Meyer, G.E., Von Bargen, K., Mortensen, D.A. (1995). Color indices for weed identification under various soil, residue, and lighting conditions. *Trans. ASABE*, *38*, 259–269.

Zarco-Tejada, P., Berjon, A., Lopezlozano, R., Miller, J., Martin, P., Cachorro, V., Gonzalez, M., Defrutos, A. (2005). Assessing vineyard condition with hyperspectral indices: Leaf and canopy reflectance simulation in a row-structured discontinuous canopy. *Remote Sens. Environ.*, *99*, 271–287.
